# Development of Thermoplastic Composite Reinforced Ultra-High-Performance Concrete Panels for Impact Resistance

**DOI:** 10.3390/ma14102490

**Published:** 2021-05-12

**Authors:** Reagan Smith-Gillis, Roberto Lopez-Anido, Todd S. Rushing, Eric N. Landis

**Affiliations:** 1 Advanced Structures and Composites Center, University of Maine, Orono, ME 04469, USA; reagan.smith@maine.edu (R.S.-G.); RLA@maine.edu (R.L.-A.); 2 U.S. Army Engineer Research and Development Center, Vicksburg, MS 39180, USA; Todd.S.Rushing@usace.army.mil

**Keywords:** composite reinforcement, ultra-high performance concrete, continuous fiber-reinforced thermoplastic composites

## Abstract

In order to improve flexural and impact performance, thin panels of steel fiber-reinforced ultra-high performance concrete (UHPC) were further reinforced with external layers of continuous fiber-reinforced thermoplastic (CFRTP) composites. CFRTP sheets were bonded to 305 × 305 × 12 mm UHPC panels using two different techniques. First, unidirectional E-glass fiber-reinforced tapes of polyethylene terephthalate glycol-modified (PETG) were arranged in layers and fused to the UHPC panels through thermoforming. Second, E-glass fiber woven fabrics were placed on the panel faces and bonded by vacuum infusion with a methyl methacrylate (MAA) polymer. Specimens were cut into four 150 mm square panels for quasi-static and low-velocity impact testing in which loads were applied at the panel centers. Under quasi-static loading, both types of thermoplastic composite reinforcements led to a 150–180% increase in both peak load capacity and toughness. Impact performance was measured in terms of both residual deformation and change in specimen compliance, and CFRTP additions were reduced both by 80% to 95%, indicating an increase in damage resistance. While both reinforcement fabrication techniques provided added performance, the thermoforming method was preferable due to its simplicity and fewer specialized tool requirements.

## 1. Introduction

The virtues of ultra-high performance concrete (UHPC) as a construction material are well documented. UHPC is generally defined as that with a compressive strength exceeding 150 MPa. The most cited traits are typically this high strength, excellent durability, and the ability to make relatively thin sections [1,2,3,4]. The material obtains its high strength through a combination of a low water-to-cement ratio and small aggregate size with optimized particle packing. While the UHPC cement matrix is extremely brittle, the incorporation of steel and other non-metallic fibers can lead to an extremely tough, even strain-hardening composite [5,6].

Because of this high toughness, fiber-reinforced UHPC quickly became a material of interest for resisting impact loads, and its merits were quickly established for a range of applications and UHPC systems [7,8,9,10,11,12,13,14,15,16,17,18]. Studies consistently show the high energy dissipation capacity of UHPC due to the interaction of the high-strength matrix with ductile steel fibers. While fibers nominally increase the initial stiffness, they significantly increase the residual capacity after impact. For impact loads, the already high compressive strength is enhanced by confinement effects that not only add strength and ductility to the UHPC matrix, but the compression field also enhances the resistance of fiber pullout [19]. For thin panels, failure is characterized by localized cracking and spalling around the impact zone [9,10,14].

Despite the numerous advantages, UHPC still has limitations with respect to low velocity impact loads. As articulated by Renade et al. [20], severe spalling can occur due to reflected tensile waves, adding to the size of the damage zone. For small impactors, the failure mode can shift from flexure to punching shear, which leaves zones of little to no structural resilience.

Efforts to reduce the effects of spalling and punching shear in concrete panels led the U.S. Army Engineer Research and Development Center (ERDC) to develop the Modular Protective System (MPS) [21], in which thin concrete panels were covered with fiber-reinforced polymer (FRP) skins. In such a system, the FRP enhances the blast and ballistic performance of the concrete core by providing additional flexural reinforcement for blast resistance and providing impact side reinforcement for slowing projectiles, in addition to opposite side reinforcement to catch any spalling concrete. The research described in this paper is an attempt to improve on the MPS technology, partly by replacing the thermoset FRP with a thermoplastic, and partly by improving the bond with the concrete.

Applications of externally bonded FRP composites have become fairly common both as supplemental reinforcement, but also as a repair method [17,22,23,24]. Typical cited advantages of FRP reinforcements are their relative light weight and tailorable properties. In applications of FRP reinforcements to concrete structures, the resin system used for the composite matrix is typically a thermoset polymer due to the generally superior strength and stiffness properties when compared to thermoplastic resins. However, thermoplastic resins were of interest here for several reasons, including rapid fabrication, toughness, ability to weld, and recyclability. To date, the applications of continuous fiber-reinforced thermoplastic composites (CFRTP) have been limited, with much of the focus on thermoplastic composites reinforcing bars due to their ability to be bent in the field [25,26,27].

In the work described in this paper, small steel fiber-reinforced UHPC panels were fabricated and externally reinforced with continuous fiber-reinforced thermoplastic (CFRTP) skins using a thermoplastic resin for both the composite matrix and the bonding agent between the concrete and the CFRTP. The goal of the research was to examine the feasibility of such a system to improve low velocity impact performance. As part of this feasibility study, two different thermoplastic processing techniques were applied. The first consisted of thermoforming previously produced CFRTP sheets to the concrete under pressure, while the second consisted of a vacuum-assisted infusion with in situ polymerization. The feasibility assessment intended to answer the following questions: (1) what are the improvements in damage resistance under low velocity impact loads? and (2) what are the relative merits of the different thermoplastic fabrication techniques? An additional item for consideration in this study was the use of cellulose nanofibrils (CNFs) as a secondary internal reinforcement for the UHPC. CNFs as a concrete admixture are gaining interest for both short term and long term properties [28,29,30]. They are relevant here because they have been shown to specifically improve the toughness of the UHPC matrix used in this study [31]. Thus a third question to be answered by this study is whether the CNF inclusion provided a measurable improvement in impact performance.

The experimental program detailed below was developed to answer these questions and to perhaps suggest strategies for further improving fabrication techniques as well as performance characteristics. The novelty in the work is the the use of thermoplastic composites as both FRP resins and bonding agents for the UHPC reinforcement.

## 2. Materials and Methods

### 2.1. Ultra-High Performance Concrete

The concrete used in this work can be classified as a UHPC due to its very high compressive strength (exceeding 200 MPa). The mix used was intended to mirror the properties of “Cor-Tuf”, a UHPC mix developed by ERDC [32]. Table 1 details the base mixture proportion used in this work. The mix features a water-to-cementitious ratio of 0.15. Workability was made possible through the combination of a specific sand size distribution (0.5 mm maximum size) as well as a superplasticizer. A second UHPC mix was also prepared with a small (0.05% by volume) dosage of cellulose nanofibrils (CNFs). Both the base and the CNF-modified UHPC mixes were reinforced with 12 mm-long by 0.20 mm-diameter brass-coated steel fibers at a volume fraction of 1.5%.

These two mixes were used to fabricate a series of 305 × 305 × 12 mm panels. The concrete was batched in a large planetary paddle mixer and placed in the panel molds using the exact weight of material required to fill the molds. The panel molds were then vibrated to consolidate and level the concrete. Specimens were placed in a 100% humidity, 25 °C fog room, demolded after 24 h, and placed back in the fog room for 5 d. Panels were then steam-cured at approximately 90 °C for 2–4 d to accelerate the curing process.

The unconfined compressive strength of the UHPC mixes was measured on 50 mm cube specimens at ages ranging from 36 to 42 days. The mean compressive strengths of the base and CNF-modified mixes were 205 and 202 MPa, respectively. There were no statistically significant effects on compressive strength due to either the length of the steam curing time or the age at testing. In particular, it should be noted that no additional strength gains were observed after the steam curing.

### 2.2. Thermoplastic Composite Unidirectional Tapes Thermoformed onto UHPC

The first thermoplastic-based reinforcement considered was a system of unidirectionally reinforced tapes employing a polyethylene terephthalate glycol (PETG) resin. PETG is amorphous, which makes it ideal for thermoforming or heated consolidation. The ability to melt and bond the thermoplastic composite tapes directly to the UHPC surface is considered an advantage of thermoforming. Consolidating the polymer tapes and UHPC required heating them together in the same oven, which presents the potential concern of UHPC expansion. Therefore, one of the most important advantages of PETG is its low processing temperature of 215 °C. Tapes with continuous E-glass fiber reinforcement and PETG manufactured by PolyOne (Avon Lake, OH, USA) were chosen for the thermoforming process. The unidirectional tape had a 58% fiber weight fraction.

**Figure 1:**
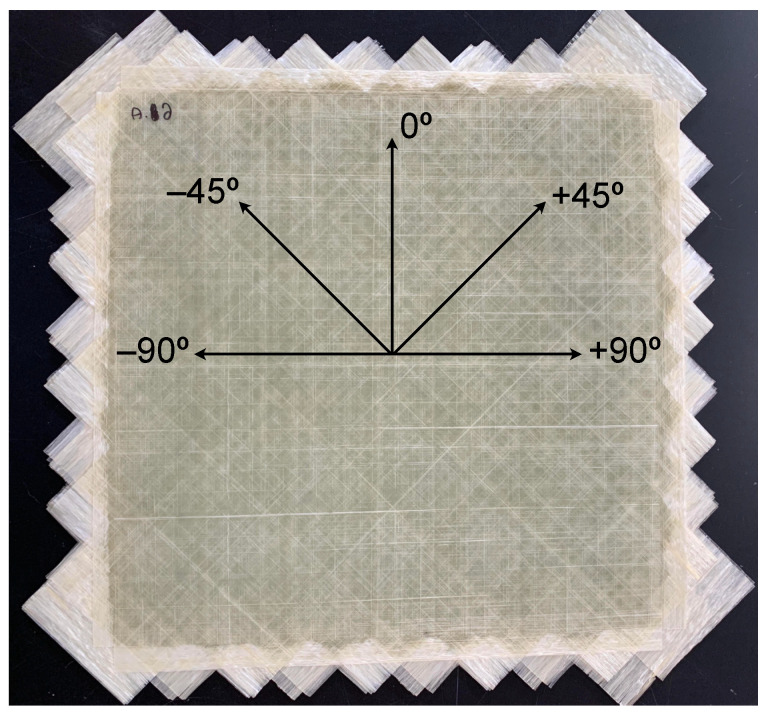
Photograph of tape layups prior to final thermoforming [38].

An eight-layer unidirectional tape layup was selected with the following fiber architecture: ${\left[0/90/-45/+45\right]}_{S}$. The fiber architecture of the unidirectional tapes was intended to increase the amount of ply delamination that occurs under low velocity impact. Delamination in multi-angle composites under low velocity impact loading was found to be more likely to occur at ply interfaces where there was a large mismatch in bending stiffness or fiber angle changes of 90° [33,34,35]. This behavior was confirmed by two studies where different fiber architectures were tested and compared in terms of their energy absorption and ply delamination under low velocity impact loading [36,37]. A photograph illustrating the tape layup on a panel is shown in Figure 1.

The thermoforming procedure for applying the thermoplastic composite tapes to the UHPC panels only allowed for consolidation on one face of the panel. Therefore, to fabricate panels with thermoplastic composite tapes on both faces, the procedure was performed twice. The material layup for consolidation was a UHPC panel, a PETG neat resin sheet, and a PETG tape multidirectional tailored blank. The PETG neat resin blank was placed between the UHPC and the PETG tape multidirectional tailored blank to create a resin-rich layer to flow into the small pores of the UHPC and create a better bond between the two materials. To allow for uniform consolidation, the panel was placed on an aluminum sheet and a silicone mat. The entire panel was placed in a 210 °C oven for 14 min. Before placement in a 50 ton press, another silicone mat and aluminum plate were placed on top of the multi-directional tailored blank. An effective pressure of approximately 70 MPa was used to press and consolidate the panel for 15 min.

### 2.3. In-Situ Polymerized Thermoplastic Composite

As an alternative to thermoforming, a second thermoplastic resin system was used that allowed the composite reinforcement to be vacuum-infused to the UHPC panel. Elium, a two-part liquid thermoplastic resin system produced by Arkema (Colombes, France), was used for this purpose. Elium is composed of between 50 and 85% methyl methacrylate (MMA) and between 10 and 50% acrylic copolymers. The system is similar to thermosetting resin systems, in that it requires an activating agent. The system has the advantage that it allows one to use traditional vacuum infusion techniques developed for thermoset resins.

Woven and stitched fabrics are typically utilized in vacuum infusion. These reinforcement types were expected to have a smaller damaged area, and therefore less energy absorption under impact loading than unidirectional reinforcement [33,37,39,40]. A previous study showed that under low velocity impact, woven fabrics increased the perforation resistance of a composite laminate while reducing the damaged area [39]. Stitched fabric has been shown to be even more effective in reducing the size of the delaminated area [40]. Based on these results, a woven 18 oz/yd^2^ E-glass fabric was selected to achieve the highest energy absorption capacity possible. The selected fabric had 4.5 to 5.5 ends per inch and 3 to 4 picks per inch; therefore, the eight layers of fabric were oriented with the ends in the following directions: ${\left[0/-45/+45/0\right]}_{S}$. The UHPC panel was placed between the two middle 0 layers; therefore, the +45 layer was always placed closest to the UHPC panel in the layup. This layup created a balanced and symmetric laminate.

Vacuum infusions were performed on a table with a precision surface. The size of the infusion was determined by the number of UHPC panels as well as the setup of the vacuum line and the resin line. Using peel o’ply and flow media, the vacuum infusion was set up and bagged. Steel fibers at the edges of the UHPC panels caused a large number of leaks in the vacuum seal. A majority of these leaks were sealed during a pressure drop test, but dry spots did occur after infusion on certain panels where leaks were not identified. These dry spots were corrected either through reinfusion or by applying Elium with a brush directly to the fabric.

It should be noted that what is presented here is an overview of specimen fabrication for both the thermoformed and infused specimens. Reference [41] provides additional details.

### 2.4. Specimens

For the experiments described here, a total of 16 305 × 305 × 12 mm composite-reinforced UHPC panels were produced; eight by thermoforming, and eight by resin infusion. Each of these groups included four base UHPC panels and four panels made of CNF-modified UHPC. Each of the 16 panels were then quartered using a diamond wet saw into 150 mm square panels for quasi-static and impact testing. UHPC and CNF-modified UHPC panels without composite reinforcements were also prepared as a control for both static and impact testing.

## 3. Experimental Methods

### 3.1. Quasi-Static Testing

**Figure 2:**
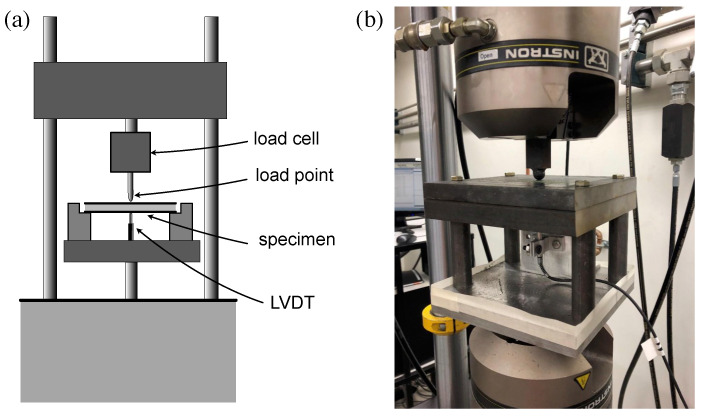
Illustration of quasi-static testing setup: (**a**) schematic, and (**b**) photograph.

As a baseline measurement prior to impact testing, composite-reinforced UHPC panel energy absorption capacity was measured through quasi-static testing. A steel test fixture was fabricated that provided simple support to the panels on all four sides. The fixture was mounted in a 100-kN servo-hydraulic load frame. Load was applied to the center of the panel with a 16 mm diameter hemispherical load head. A linear variable differential transformer (LVDT) was mounted beneath the panel to measure load-point displacement. The configuration is shown in Figure 2. Tests were conducted under position control using an actuator rate of 1 mm/min until the position difference reached 4 mm. Load, load-point displacement, and position data were recorded, and the load versus load-point displacement was plotted for each test. From each plot, peak load was noted, and the energy absorption capacity was calculated as the area under the load-displacement curve up to a two millimeter displacement.

### 3.2. Low-Velocity Impact Testing

**Figure 3:**
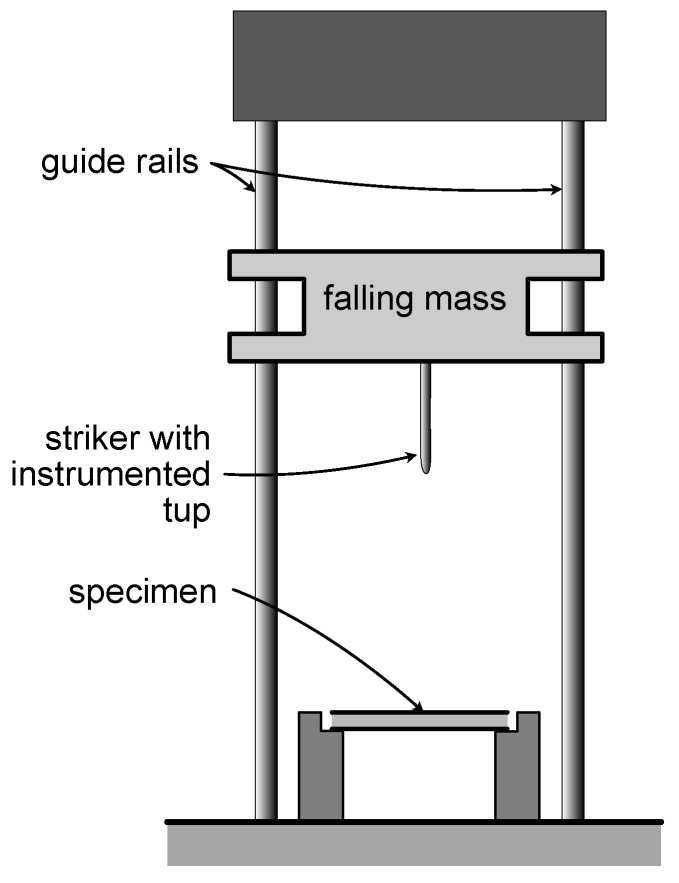
Schematic illustration of drop weight impact testing tower.

Low-velocity impact testing was performed with a drop-weight testing system (Instron 9350 CEAST (Instron, Norwood, MA, USA)), a simplified schematic of which is shown in Figure 3. As with any dynamic testing, the potential for inertial effects needed to be considered. Different approaches have been used to account for inertial effects during low-velocity impact testing of concrete [42,43]. In certain situations where the mass of the impactor is significantly larger than the mass of the specimen, the inertial effects have typically been neglected [14,44]. For the panels tested here, the mass of the impactor was more than three times larger than the mass of the heaviest specimen; thus, the inertial effects were not included in the low-velocity impact testing analysis.

The testing configuration for the impact testing was similar to the quasi-static setup. The panel specimens were simply supported on all four sides, and the impact force was provided by a 16 mm hemispherical steel tup with a built-in accelerometer. The drop tower was controlled by integrated control and data acquisition software that provided the following data for the entire test: time, impact force, impact energy, tup displacement, and tup velocity. The system was user-programed to impact the specimen at a specified energy.

**Figure 4:**
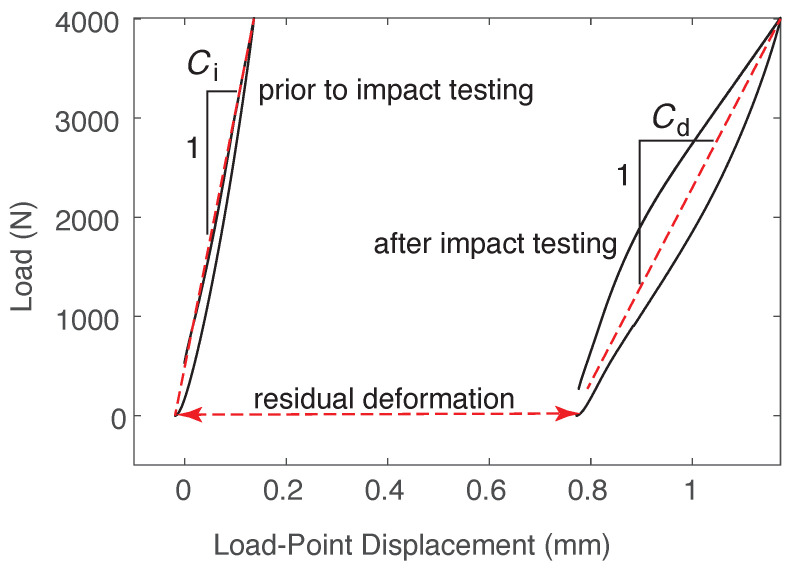
Illustration of measured parameters: initial compliance, $C_{i}$, damaged compliance, $C_{d}$, and residual deformation.

In addition to the data collected by the drop tower instrument, residual deformation and change in compliance were used to evaluate the impact performance of the panels. These parameters, illustrated in Figure 4, were measured by first inserting the panel into the servo-hydraulic load frame described in Section 3.1. The LVDT was zeroed, and a load of 4 kN was applied (2 kN for the panels without composite reinforcement). The initial compliance was taken as the inverse of the slope of the load versus load-point displacement plot. After impact testing, the damaged specimen was put back into the static frame. The initial LVDT reading was taken as the residual deformation. The specimen was then loaded to 4 kN and unloaded. Due to significant hysteresis in the damaged specimen, damaged compliance was measured using the two points: initial (residual) deformation (at zero load) and the deformation at 4 kN, as illustrated in Figure 4. Change in compliance was simply calculated as $C_{d}-C_{i}$.

Low-velocity impact tests were conducted as follows. The specimen was secured in the steel base, and the drop tower was programed to perform a single impact on the specimen. Based on the energy absorption measurements from the quasi-static panel tests, the drop tower was initially set up to impact the specimens at 16 J. This impact energy was used for the unreinforced specimens and two specimens of each composite-reinforced group. Composite-reinforced groups each had two specimens impacted at 24, 32, and 40 J. An anti-rebound system was employed to assure a single impact. Data from the drop tower were analyzed to compare the peak impact force and the maximum deflection of the tup during the impact. The area under the load-deflection curve was also calculated to estimate the amount of energy imparted to the specimen during the impact.

## 4. Results

### 4.1. Quasi-Static Test Results

**Figure 5:**
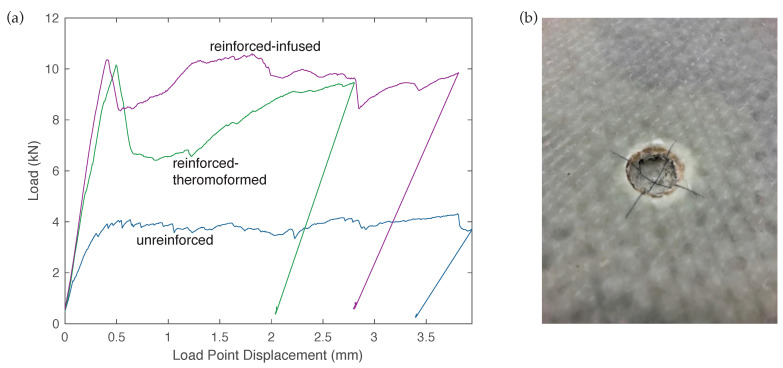
Example results: (**a**) quasi-static load-deformation plots for three different specimen types, and (**b**) example CFRTP load point damage (**b**).

Quasi-static load-deformation data were recorded for 42 panel tests. Example plots for the different specimen types are shown in Figure 5a. The plots illustrate the typical behavior of all panels. All panels showed good ductility, but the CFRTP-reinforced panels showed much higher load capacities. The CFRTP-reinforced specimens showed several behaviors that distinguished them from the UHPC-only panels. Reinforced specimens made both by thermoforming and infusion had an initial peak, followed by a load drop, and then a recovery. This reflects a damage mode in which the hemispherical load head penetrated the CFRTP (Figure 5b), but bottomed out against the UHPC leading to the recovery. This behavior was also reflected in the different maximum displacements. The test was stopped when the instrument stroke reached 4 mm. The displacement shown in Figure 5a was based on the LVDT mounted beneath the panel, so that any CFRTP penetration by the load-head was not reflected in that displacement. It should be noted that the UHPC specimens without CFRTP reinforcement also showed localized penetration at the load point, but it was not as pronounced as was found with the CFRTP specimens.

**Figure 6:**
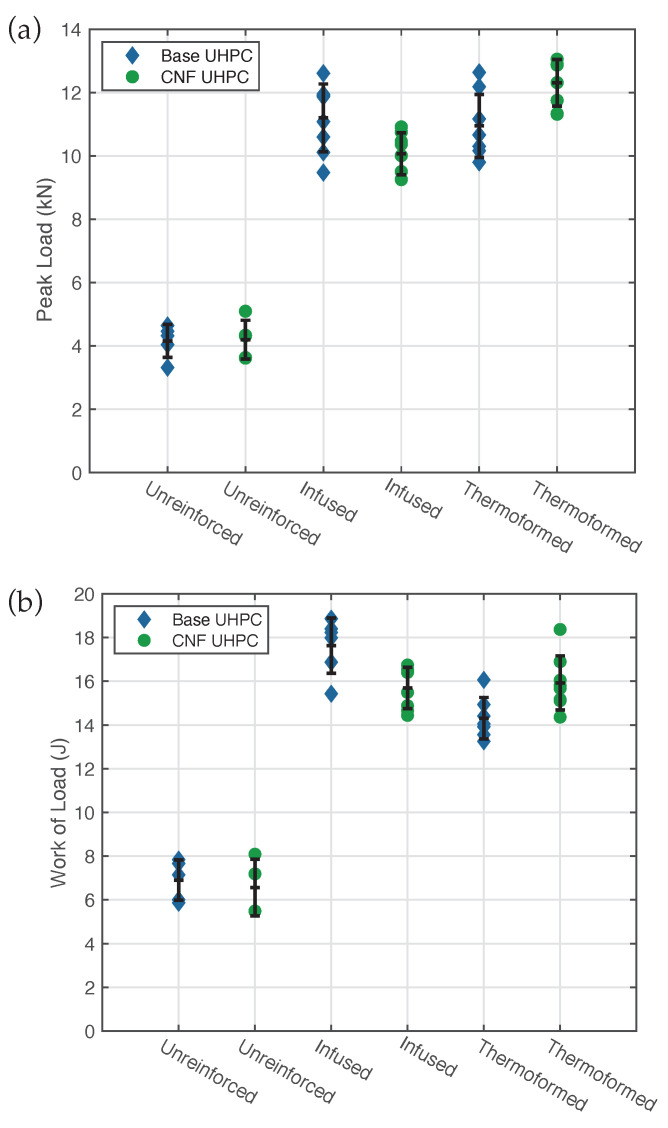
Plots of (**a**) peak quasi-static peak load, and (**b**) work of load up to 2 mm deflection, for different panel types.

Each load-deformation plot was evaluated by determining the peak load and by calculating the work of the load up to a 2 mm load-point displacement. Peak loads for all specimens are shown in Figure 6a. The error bars represent one standard deviation above and below the mean value for peak load. Minimal variation between UHPC panels with and without CNF was observed, suggesting that the CNF provided little to no additional strength. As expected, the addition of CFRTP reinforcements to the UHPC significantly increased the load carrying capacity. The infused specimens showed a 155% increase over the unreinforced specimens, whereas thermoformed specimens showed a slightly larger 178% increase in mean peak load. Similar improvements were seen with the net work of load up to a deflection of 2 mm, as shown in Figure 6b.

Quasi-static panel tests on the unreinforced UHPC specimens demonstrated results similar to those suggested by Ranade et al. [20], who tested ultra-high performance concrete for impact resistance. They described a UHPC panel failure due to brittle punching shear with a small diameter loading head. When the unreinforced UHPC panels were subjected to the 16 mm ball bearing loading head, a punching shear failure was commonly induced. The thermoplastic composite-reinforced specimens failed through radial debonding of the rear face composite. When the composite was removed from the rear face of the UHPC after the test, there was evidence of punching shear failure in the UHPC. In those specimens, the cracking was not as severe as the unreinforced specimens, which indicated the severity of the punching shear failure was reduced by the addition of the CFRTP skins.

### 4.2. Low-Velocity Impact Testing

The drop tower instrument used in this work recorded force and tup displacement at small time increments. Tup displacement was zeroed on the specimen surface prior to impact. The drop tower instrument was set up to strike the panels at four different impact energies; 16, 24, 32, and 40 J. In order to confirm these impact energy values, the impact load vs. deflection plots recorded by the instrument were integrated to estimate the energy. For the 16 J impact, the mean area under the load-deflection curve was 19 J, suggesting that there were likely inertial effects unaccounted for. The specimens impacted with 24, 32, and 40 J also exhibited greater areas under the load-deflection curves with mean areas of 29, 38, and 44 J, respectively.

**Figure 7:**
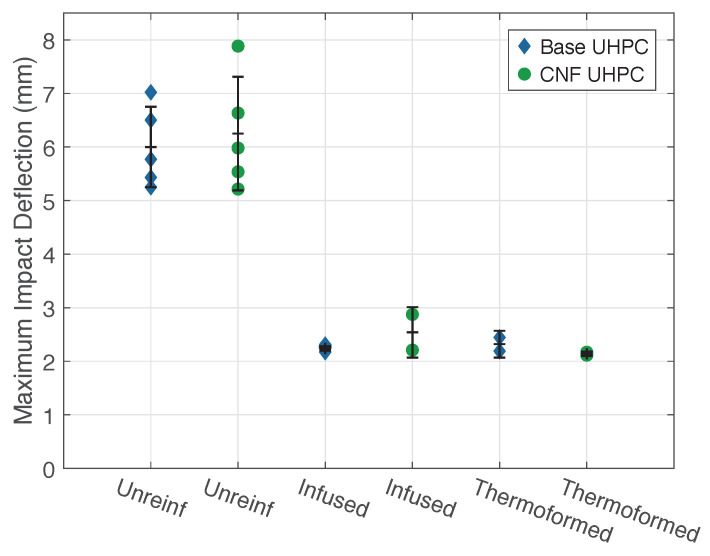
Plot of maximum panel deflections, impact energy of 16 J.

Figure 7 shows the maximum tup/panel deflection during a 16 J impact for all specimen types. As expected, the unreinforced UHPC panels had a significantly greater deflection during impact than the CFRTP-reinforced panels. Upon post-impact examination, the unreinforced UHPC panels had significant radial cracking on the rear face of the panels. The unreinforced specimens had a broad distribution of results, likely due to the random orientation of the steel fibers [8,45,46]. The plot shows the significant reduction in the maximum deflection when the CFRTP is added. The infused reinforcement reduced the mean maximum impact displacement by 61%, while the thermoformed reinforcement was slightly more effective, reducing the mean maximum impact displacement by 64%. In all cases, the CNF addition to the UHPC mix had little to no effect, and the difference between the results of the two CFRTP reinforcements was minimal.

**Figure 8:**
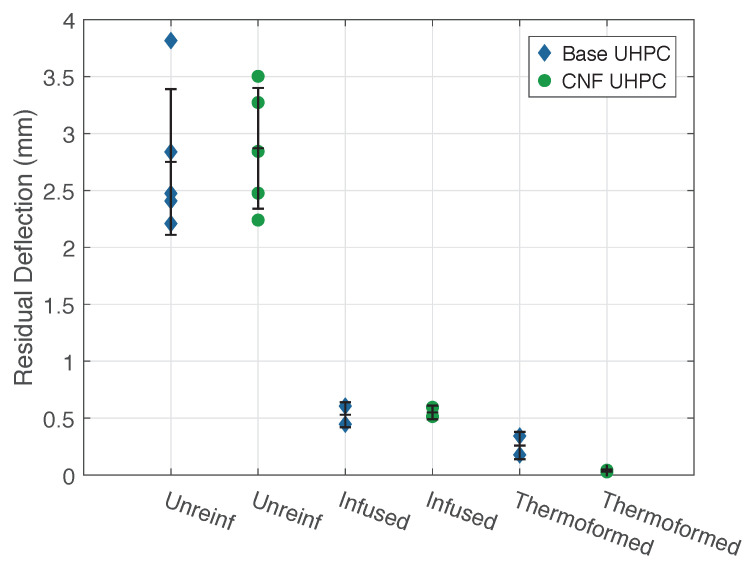
Residual deflections for panels at 16 J impact.

Residual deflection was used in this study as one measure of impact damage, illustrated in Figure 4. Figure 8 demonstrates the residual deflection of all specimen types at an impact energy of 16 J. The figure shows that specimens with CFRTP reinforcement had significantly lower residual deflections than the unreinforced UHPC panels. This was due to the brittle punching shear failure of the UHPC. The thermoplastic composite reinforcement on the rear face of the panel was effective at catching the displaced concrete, resulting in lower residual deflections. There was no significant difference between the performance of base UHPC and CNF-modified UHPC. The panels reinforced with infused CFRTP averaged an 81% reduction in residual deflection, whereas the panels with thermoformed CFRTP showed a reduction of over 95%.

**Figure 9:**
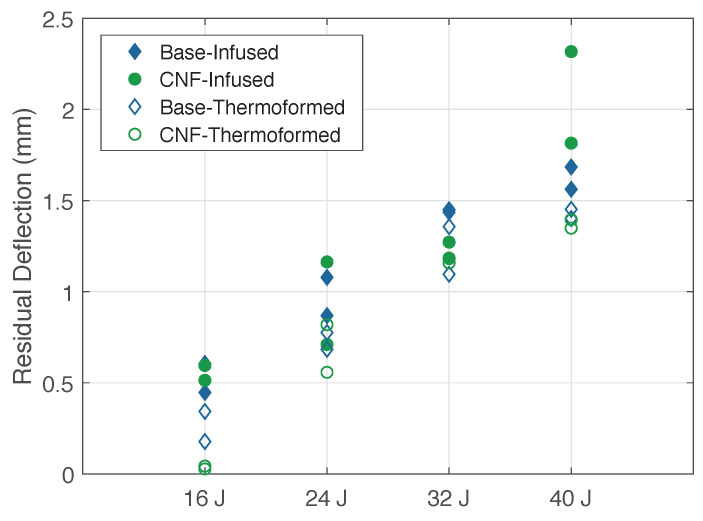
Residual deflections for CFRTP panels at different energy levels.

Figure 9 shows the residual deflection results of the CFRTP-reinforced panels at the different impact energies. As expected, residual deflection increased with impact energy. The degree of scatter tended to be dependent on degree of delamination induced by the impact load, with higher degrees of delamination leading to higher residual deflection.

The second measure of damage used in this work was the change in specimen compliance induced by the impact load, with an increase in compliance indicating an increase in damage. Panels were quasi-statically loaded and unloaded before and after impact so that the change in compliance could be determined. (Figure 4).

**Figure 10:**
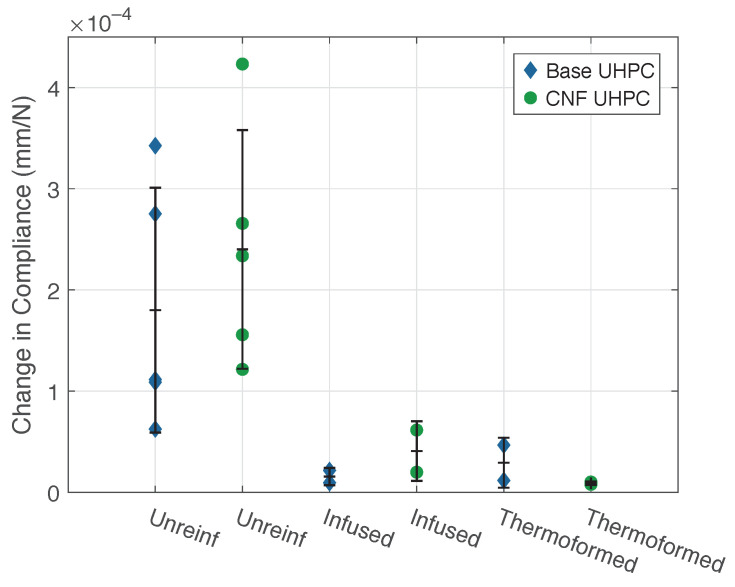
Compliance changes for 16 J impact, all panels.

In Figure 10, the change in each specimen’s compliance for a 16 J impact is shown for the different panels types. The change in compliance of the unreinforced specimens showed significant scatter for both the control and CNF-modified UHPC, indicating a wide range in the degree of damage to each specimen. Nevertheless, using mean change in compliance for the unreinforced panels, one can observe a reduction of 87% for the infused CFRTP-reinforced panels and 91% for the thermoformed CFRTP-reinforced panels. The infused panels had a mean change in compliance of $2.8\times 10^{-5}$ mm/N, while the pressed thermoplastic CFRTP-reinforced panels had a slightly lower change at $1.9\times 10^{-5}$ mm/N.

**Figure 11:**
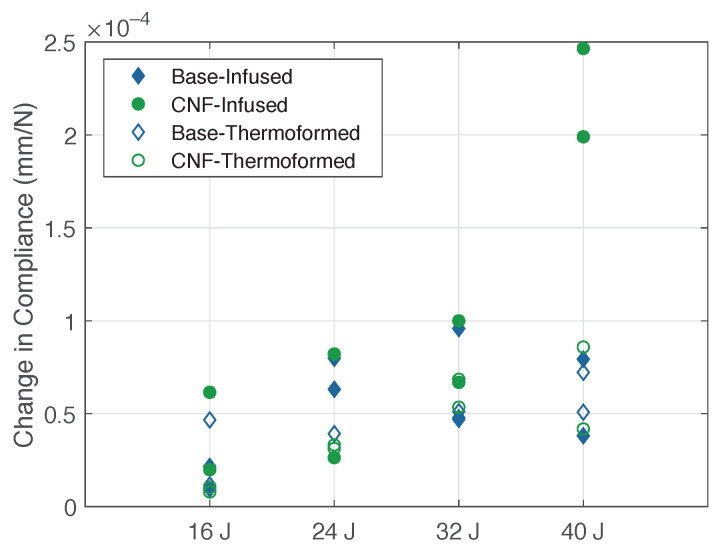
Compliance changes for CFRTP panels, all energies.

Figure 11 shows the change in compliance for the CFRTP-reinforced UHPC panels at different impact energies. The base UHPC panels suffered the most damage at 24 and 32 J, whereas the CNF-modified panels showed a change in compliance proportional to the impact energy. It should be noted that the large changes in compliance at 40 J for the infused CFRTP CNF-modified UHPC panels were due to delamination of the rear face thermoplastic. The thermoformed reinforced control UHPC panels had consistent values at 24, 32, and 40 J.

**Figure 12:**
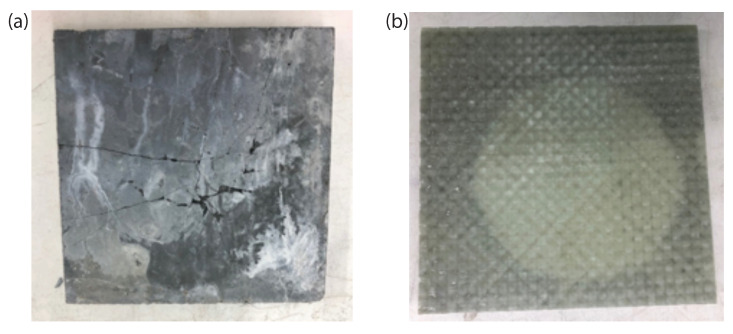
Photos illustrating (**a**) unreinforced punching shear, and (**b**) radial delamination of infused CFRTP reinforcement.

The failure modes of the unreinforced UHPC panels and the thermoplastic composite-reinforced UHPC panels during impact were similar to the failure modes during the quasi-static panel testing. In Figure 12a, the start of a punching-shear failure can be seen in an unreinforced UHPC specimen, while Figure 12b shows a radial delamination in an infused CFRTP-reinforced panel. This damage pattern was common among the infused panels. The opaque nature of the thermoformed specimens made it more difficult to see delamination, although a “tap test” showed that delamination did occur.

**Figure 13:**
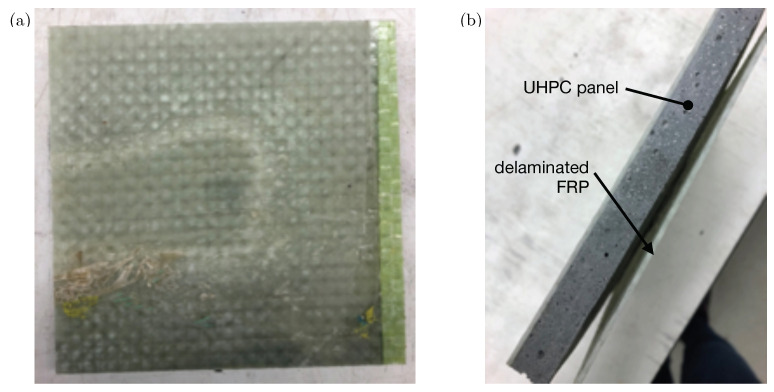
Photos illustrating (**a**) delamination of infused specimen containing “dry spot” during fabrication, and (**b**) residual deformation and separation of CFRTP from UHPC after delamination.

As previously stated, the CNF-modified UHPC panels reinforced with infused CFRTP delaminated when they were impacted at 40 J. Figure 13a shows the reinfused section on the rear face of one of these panels. The dry spot from the initial infusion followed by the reinfusion of the panel was suspected to be the reason for the complete delamination. Typically, the reinforced specimens only suffered radial delamination, but in the case of two specimens, a complete delamination occurred where the infused CFRTP separated from the UHPC. The separation of the infused thermoplastic composite is shown in Figure 13b. No thermoformed specimens suffered complete delamination.

## 5. Discussion

The experimental results bring up several issues for discussion. First, it is clear that the addition of thermoplastic composite skins to UHPC panels significantly increased the impact resistance of the UHPC. It should be noted that all of the starting UHPC panels were of the same thickness, so the application of composite reinforcements added to the overall panel thickness relative to the unreinforced UHPC panels. Clearly some of increase in perforation resistance could be simply attributed to the additional material; however, this study did not attempt to resolve it quantitatively. Future work will include testing of the CFRTP materials alone in order to better determine whether the performance of the reinforced UHPC panels is the sum of the components, or whether there are synergistic effects. In terms of the measured damage indicators of residual deformation and change in compliance, all reinforcements improved performance. However, within the range of experimental scatter, it is more difficult to make clear distinctions between both the role of CNF in the UHPC and the two different thermoplastic reinforcement types.

In the quasi-static tests of the specimens tested, CNF appeared to have the effect of reducing peak load and work load in the infused panels, while increasing the peak load in the pressed panels. In the impact tests, the addition of CNF to the UHPC mix did not have a statistically significant effect. Since the addition of CNF to the UHPC mix complicated the preparation of the UHPC panels, we would conclude from these experiments that the real additional cost in materials and fabrication is not worth the minimal if any benefit.

Regarding the two different thermoplastic composites, there were some observed differences between the results of the quasi-static tests and the impact tests. In the case of the quasi-static peak load, it should be noted that since there was some penetration of the CFRTP reinforcement by the hemispherical loading head, peak force can be thought of as a combination of panel strength and CFRTP hardness. Regardless, combining base and CNF modified UHPC panel types, there were no statistically significant differences between the mean peak load or the mean work load for the two reinforcement types. However, in the impact results, the thermoformed CFRTP-reinforced panels tended to slightly outperform the infused CFRTP-reinforced panels, particularly at the higher impact energies.

While there were small differences in the performance of the different panels, we submit here that the significance of these differences are trivial compared to the issues associated with fabrication and/or installation of these types of reinforced panels. For the case in which panels would be reinforced prior to service, the thermoformed panels hold significant advantages both for small batch and large-scale production. The resin infusion process, while a common method of composite manufacture, is time- and labor-intensive compared to the thermoforming. Additionally, the thermoforming can more readily be scaled for rapid large scale production. The practical limit on thermoforming is specimen size, because larger panels require larger presses. There is no fabrication size limit for infusion, so long as a large tool (in this case, a flat surface) is available.

Regarding delamination of CFRTP, it is likely that boundary effects played a role. While all panels were simply supported, under quasi-static testing, the reaction forces acting on the panels at the supports put the CFRTP bond in compression, which helped keep the bond from failing. This particular boundary effect was assumed to play a smaller role during impact as a flexure wave passing through the panel could have had the effect of reducing or eliminating that compressive stress during the course of the test.

Finally, as alluded to in Section 4.2, there were differences between the programed impact energy (e.g., *mgh*), and the impact energy calculated from the drop weight force and displacement data. Specifically, impact energy calculated from the load and displacement data was three to six joules higher than the impact energy specified by the user, which points toward an effect of inertial forces. We acknowledge this potential measurement error, and we will make appropriate changes to our test setup to better account for inertial effects in future testing. However, we submit that the basic conclusions of these experiments would not change.

## 6. Conclusions

The research described in this paper was aimed at testing the feasibility of thermoplastic CFRTP reinforcing for thin concrete panels subjected to low velocity impact loads. Feasibility was established through a specimen fabrication and testing program, which was intended to determine how well the CFRTP reinforcement would enhance the low velocity impact performance of the UHPC. The results of the experiments showed significant (150–180%) improvements in quasi-static strength and toughness. Impact performance was measured using two parameters: residual deformation and change in specimen compliance. The results showed 60% reductions in residual deformation, and 80–95% reductions in compliance change under impact loading. An additional aspect of the feasibility study was a comparison of the two specimen fabrication methods. While both methods produced similar performance gains, the thermoformed CFRTP panels were far easier to produce than those produced by resin infusion. The research results suggest that the next development step will be towards better characterization of the concrete–CFRTP bond. Such characterization, along with an appropriate material model, will allow us to refine both CFRTP fiber layups and processing parameters for additional performance improvements.

## Figures and Tables

**Table 1:** UHPC constituents and proportions.

Material	Proportion by Weight
Type I/II Portland Cement	0.384
Silica Fume	0.067
Silica Sand	0.475
Water	0.068
Superplasticizer	0.006

## Data Availability

The data presented in this study are available on request from the corresponding author.
